# Long-Term Effectiveness of a Clinician-Assisted Digital Cognitive Behavioral Therapy Intervention for Smoking Cessation: Secondary Outcomes From a Randomized Controlled Trial

**DOI:** 10.1093/ntr/ntac113

**Published:** 2022-04-26

**Authors:** Jamie Webb, Sarrah Peerbux, Alfonso Ang, Sarim Siddiqui, Yusuf Sherwani, Maroof Ahmed, Hannah MacRae, Hannah Puri, Azeem Majeed, Suzette Glasner

**Affiliations:** Department of Clinical Affairs, Digital Therapeutics, Inc., San Francisco, CA, USA; School of Medicine, Imperial College London, London, UK; Department of Clinical Affairs, Digital Therapeutics, Inc., San Francisco, CA, USA; Integrated Substance Abuse Programs, David Geffen School of Medicine, UCLA, Los Angeles CA, USA; Department of Clinical Affairs, Digital Therapeutics, Inc., San Francisco, CA, USA; Department of Clinical Affairs, Digital Therapeutics, Inc., San Francisco, CA, USA; Department of Clinical Affairs, Digital Therapeutics, Inc., San Francisco, CA, USA; Department of Clinical Affairs, Digital Therapeutics, Inc., San Francisco, CA, USA; Department of Clinical Affairs, Digital Therapeutics, Inc., San Francisco, CA, USA; School of Medicine, Imperial College London, London, UK; Department of Clinical Affairs, Digital Therapeutics, Inc., San Francisco, CA, USA; Integrated Substance Abuse Programs, David Geffen School of Medicine, UCLA, Los Angeles CA, USA

## Abstract

**Introduction:**

This study evaluated the secondary effectiveness outcomes for Quit Genius, a digital clinician-assisted cognitive behavioral therapy (CBT) intervention for smoking cessation.

**Methods:**

Adult smokers (*N* = 556) were randomly assigned to Quit Genius (*n* = 277), a digital, clinician-assisted CBT intervention or very brief advice (VBA) to stop smoking, an evidence-based, 30-s intervention designed to facilitate quit attempts, coupled with referral to a cessation service (*n* = 279). Participants were offered combination nicotine replacement therapy (patches and gum) tailored to individual nicotine dependence. Analyses (*n* = 530), by intention-to-treat, compared Quit Genius and VBA at 4, 26, and 52 weeks post-quit date (QD). The primary outcome was self-reported 7-day point prevalence abstinence (PPA) at 4 weeks post-QD. Consecutive 7-day point-prevalence abstinence, defined as abstinent at two or more consecutive timepoints, was examined at weeks 26 and 52 to indicate long-term effectiveness. Abstinence was verified using a random sample of participants with carbon monoxide breath testing of <5 parts per million (*n* = 280).

**Results:**

Self-reported consecutive 7-day PPA at weeks 26 and 52 for Quit Genius was 27.2% and 22.6%, respectively, compared with VBA which was 16.6% and 13.2% (RR = 1.70, 95% CI, 1.22-2.37; *p* = .003, 26 weeks; RR = 1.71, 95% CI, 1.17–2.50; *P* = .005, 52 weeks). Biochemically verified abstinence was significantly different at 26- (*p* = .03) but not 52 weeks (*p* = .16). Quit Genius participants were more likely to remain abstinent than those who received VBA (RR = 1.71, 95% CI 1.17–2.50; *p* = .005).

**Conclusions:**

This study provides secondary evidence for the long-term effectiveness of Quit Genius in comparison with VBA. Future trials of digital interventions without clinician support and comparisons with active treatment are needed.

**Implications:**

The long-term effectiveness of clinician-assisted digital smoking cessation interventions has not been well studied. This study established the long-term effectiveness of an extended CBT-based intervention; results may inform implementation of scalable approaches to smoking cessation in the health system.

## Introduction

Smoking is the leading cause of preventable death worldwide, causing over 8 million deaths annually^1^ with a total annual global economic cost exceeding $1.4 trillion.^2^ Despite advances in pharmacological and behavioral smoking cessation treatment approaches, even when evidence-based treatments are combined with best practice guidelines, long-term abstinence rates remain relatively low, frequently falling below 20% at 1 year post-intervention.^3,4^ Given that tobacco addiction is characterized by a chronic and relapsing clinical course, longer term treatment models that support multiple transitions between relapse and recovery may optimize outcomes.^5^ Though traditional smoking cessation support involves low-intensity motivational advice, studies combining pharmacological smoking cessation treatment with extended counseling ranging from 6^5,6^ to 12 months in duration^7^ have produced superior clinical outcomes. Nevertheless, utilization rates of evidence-based smoking cessation treatment programs are astonishingly low, with data revealing that the majority of quit attempts made by daily smokers were unaided. The use of digitally delivered smoking cessation approaches is a promising pathway to provide effective support to populations that may otherwise be difficult to reach and engage.^8^

Progress in mobile technology has led to the proliferation of mobile health (mHealth) approaches to smoking cessation, conferring many advantages over face-to-face approaches, including low cost, greater accessibility, customizable features and scalability. Nevertheless, according to a recent systematic review, among the 50 most highly recommended apps suggested by leading app stores, only 4% of apps were found to be effective through comparative assessment,^9^ and a more recent review found that the majority (95%) of existing mHealth apps did not meet high quality standards, due to low utilization of evidence-based treatment strategies.^10^

Following evidence-based treatment guidelines,^4,11^ our group developed Quit Genius, a digital clinician assisted intervention combining pharmacotherapy with cognitive behavioral therapy (CBT) within an extended treatment model. Quit Genius is a 52-week digital clinician-assisted CBT tobacco cessation program combining digital CBT with coaching support delivered asynchronously within the app. Concurrent access to nicotine replacement therapy was provided to each participant. We recently reported the primary outcome of the RCT comparing Quit Genius to Very Brief Advice (VBA), in which 7-day point prevalence abstinence (PPA) of 44.5% at 4 weeks post-quit-date was observed, verified by expired CO <10 ppm.^12^ In the present study, we examined the effect of Quit Genius on longer-term, secondary outcomes through 52 weeks post-quit date (QD). To achieve this, we evaluated 7-day PPA at 4-, 26-, and 52 weeks post-QD, emphasizing consecutive 7-day PPA (i.e., 2 or more consecutive 7-day PPA observations). The impact of Quit Genius, relative to the control condition on other secondary outcomes including sustained abstinence, quit attempts, psychological well-being, and self-efficacy was also examined.

## Methods

### Study Design and Participants

We conducted a single-blinded, two-arm parallel design, randomized controlled trial (1:1 allocation ratio), with 4-, 26-, and 52-week follow-up. The trial was registered in the International Standard Randomized Controlled Trial Number database (https://www.isrctn.com/ISRCTN65853476) on December 18, 2018. This study complied with the Declaration of Helsinki and ethical approval was granted by the Health and Social Care Research Ethics Committee A (reference 18/NI/0171).

Participants were recruited via social media and referrals from primary care practices in the United Kingdom between January and November 2019. Participants were directed to a study website, where they were provided with study information and prompted to complete pre-screening questions. They were then invited to an in-person baseline assessment in which eligibility was confirmed. Subsequently, informed consent was obtained.

Individuals were eligible to participate if they were adult smokers (aged ≥18), smoked >5 cigarettes a day for the past year, had the required mobile phone functionality (>5th generation iPhone or version *>*18 Android) and were not using any other form of behavioral or pharmacological smoking cessation support. Exclusion criteria included not speaking English, pregnancy, COPD, currently taking psychiatric medication, and any serious health conditions that would hinder completion of the study procedures. In total, 556 participants consented to participate, completed screening, and were randomly assigned to Quit Genius or the control condition.

### Randomization and Blinding

This study was a single-blinded randomized controlled trial. Participants were randomized with an allocation ratio of 1:1 (treatment:control) using a block size of 4 participants. Researchers were blinded to treatment allocation until after randomization had been performed.

### Combination NRT

All participants were offered nicotine replacement therapy (NRT) comprising patches and/or gum for 12 weeks, with the first 2-weeks supplied at the baseline visit. Participants who smoked within the first 30 min of waking were offered 24-h 21 mg patches; otherwise, they were offered 16-h 25 mg patches. Participants were given patches in steps 1–3 (4 weeks per step); 24-h patches (21, 14, and 7 mg); 16-h patches (25, 15, and 10 mg). In addition to patches, participants who smoked *>*20 cigarettes a day were offered 4 mg gum. If participants smoked <20 they were offered 2 mg gum. Participants were also allowed to use other forms of oral NRT.

### Treatment Intervention: Quit Genius

Quit Genius^13^ was a 52-week digital clinician-assisted CBT intervention. Quit Genius comprised a smartphone app with self-guided CBT content, coupled with a quit coach who provided asynchronous messaging to reinforce CBT skills. In addition, Quit Genius utilized components that have demonstrated efficacy in promoting smoking cessation, including encouraging medication adherence, goal setting and self-monitoring.^10,14–16^ The app collected user data that tailored the pace (i.e., speed at which a participant navigated through the CBT) and content to each participant, based upon app utilization and other individual variables including self-reported reasons for quitting and quit-date. Participants could reset QDs as needed.

Participants were prompted to complete consecutive self-paced CBT and motivational steps, drawing from the extended CBT model developed by Hall et al.^7^ The following content areas were included: (1) motivation, (2) dependence and withdrawal, (3) social support, (4) identifying triggers, (5) coping with cravings, (6) cognitive restructuring, (7) managing negative affect, (8) weight gain, and (9) relapse prevention planning. Further detail surrounding each content area is provided below:

(1) *Motivation*: based on the principles of motivational interviewing, participants were prompted to examine their readiness to quit, complete motivational exercises within the app such as listing their individual reasons for quitting, and considering the pros and cons of continuing to smoke versus quitting, with reinforcement of the content in interactions with their quit coach. Psychoeducation concerning health consequences of smoking, costs of smoking, and health and financial benefits of quitting was provided both by the quit coach and in the form of digital interactive exercises including quizzes and tailored feedback as the individual accrued hours and days smoke-free post-QD.
(2) *Dependence and withdrawal*: Psychoeducation was provided concerning signs of dependence on nicotine. Participants were encouraged to monitor their withdrawal signs and were provided with strategies for coping with specific symptoms within the app, and reinforced by their quit coach.
(3) *Social support*: Participants examined triggers in their existing social network, learned assertive communication techniques to help them resist pressures to smoke, and were encouraged to expand their network of nonsmokers.
(4) *Identifying triggers*: In-app exercises supported participants in identifying their unique smoking triggers.
(5) *Coping with cravings*: Participants had access to a “Craving Toolbox” within the app, comprising audio mindfulness, meditation and breathing exercises, CBT content including strategies for coping with stress and negative affect, managing temptations in social situations, and the role of general care practices in preventing relapse, such as increasing physical activity. The quit coach also provided support to reinforce coping skills practice.
(6) *Cognitive restructuring*: In-app exercises, reinforced by the quit coach, targeted identification of irrational thoughts about smoking (e.g., “I really need a cigarette”), with strategies to replace thoughts that could arise during cravings with alternatives (e.g., “I can resist this one cigarette”).
(7) *Managing negative affect*: Content within the app related to mood and anxiety management focused on monitoring smoking behavior in relation to affective states, developing an understanding of how mood states influence their smoking frequency and quantity, employing cognitive restructuring when needed to cope with negative affect, increasing pleasant activities, and monitoring the impact of pleasant activities on mood.
(8) *Weight gain*: As a means of both weight control and mitigating depression, increasing physical activity was emphasized both in the app content and coaching interactions, through goal-setting, scheduling, problem-solving and monitoring and reinforcing progress.
(9) *Relapse prevention planning*: Throughout the app and with support from the quit coach, relapse prevention planning comprised reviews of core therapeutic skills including recognizing triggers, coping with cravings, using and adhering to NRT, optimizing social support, and utilizing self-help resources.

Content was divided into two stages: “Essentials” and “Sustain.” In the Essentials stage, the user was prompted to complete various digital psychoeducation and motivational exercises concerning the behavioral and NRT components of treatment. Participants could revisit and access the essentials content at any time, and depending on readiness to quit, the “Essentials” intervention phase could be extended by postponing or resetting their QD. The ‘Sustain stage, which became accessible to the participants post-QD, focused on supporting long-term abstinence and once unlocked, users could revisit the content as needed.

A “Quit Coach”, qualified by the National Centre for Smoking Cessation and Training, provided personalized CBT-based support via the in-app chat and phone, with a 30-min phone call at baseline, discussing their individualized quit plan, and methods of using NRT. Subsequently, interactions transitioned to the in-app chat platform. Features included: progress monitoring, including feedback concerning health improvements and cost savings. Push notifications consisted of personalized goals, NRT reminders, coach messages, psychoeducation, and motivational messages. Psychoeducation messages (e.g., those reinforcing health achievements) were sent at pre-specified intervals post-QD, for example, “Your blood pressure, heart rate and the temperature in your hands and feet have returned to normal,” and “Your risk of heart disease is about half compared with a person who is still smoking”. The schedule of push notifications was tailored according to individual factors including: NRT use, cigarettes smoked per-day, money spent on smoking and responsiveness to coach messages. On the QD, push notification frequency was at its highest, and between six and 10 push notifications were sent at pre-specified intervals throughout the day. In the week post-QD, two to three notifications were sent per day. Between weeks 2 and 4 post-QD, one push notification was sent daily. Beginning at week 5, the frequency of push notifications gradually tapered, from every other day to monthly through 12 months post-QD. Coach messages containing standardized content to celebrate the QD, remind participants of instructions around NRT, check in concerning progress, and reinforce in app CBT skills practice, were delivered 3 days pre-QD, on the QD, and 3 days, 2-, 3-, and 4-weeks post-QD. Following the 4-week post-QD coach message, the frequency of coach messages tapered and tailored to individual needs throughout the remainder of treatment (see supplementary table for examples of coach messages and push notifications).

### Control Intervention: VBA

VBA is a simple form of advice designed to increase referrals to smoking cessation services. VBA follows the structure of “Ask” patients about their tobacco use, “Advise” them that the best method of quitting is with a combination of medication and behavioral support, and “Act” by supporting them with making a quit attempt using available cessation support. Participants receiving VBA were advised to contact their local stop smoking service to access behavioral support and pharmacotherapy to help them quit smoking. Research assistants who had completed the NCSCT smoking cessation course were trained to deliver the VBA, face-to-face intervention, per NCSCT guidelines (https://elearning.ncsct.co.uk). The VBA session was up to 1 h in duration, with 10 min dedicated to advising engagement in a smoking cessation program, and the remainder of the session comprising questions and answers and searching for a personalized local list of smoking services. At the conclusion of the session, participants were reminded to contact their preferred stop smoking service. Among control group participants allocated a CO device, a mobile-app (ASH-app) was provided to enable them to view their CO results. The control group mobile-app was only used in conjunction with the CO device and contained no counseling content.

### Remote Biochemical Verification of Smoking Abstinence

A random sample of ~50% of the participants in each treatment group were issued a carbon monoxide (CO) monitor for biochemical verification of self-reported smoking status (Smokerlyzer, coVita Inc.). Participants were selected for remote video-assisted CO testing using block randomization. CO levels were submitted by participants at 4-, 26-, and 52-week post-QD. The CO devices connected via headphone/charging slot of participants’ smartphones, and were only used in conjunction with the Quit Genius and the Analyzing Smoking Habits (ASH) app, which was used to visualize CO measurement data. At each of the follow-up visits, participants were asked to submit a reading from their device via phone or online to validate their self-reported abstinence status. CO readings <5 ppm were considered indicative of abstinence. The percentage of self-reported abstinence matching CO <5 ppm was calculated from the readings available at each time point and not from all of those allocated to a CO device.

### Outcome Measures

Outcome measures were collected via phone or online at baseline, 4-, 26-, and 52-week post-quit date (post-QD). Participants received £10 to offset travel costs and were compensated for each follow-up data collection visit as follows: £10 (26 weeks) and £20 (4- and 52- week follow-ups). The pre-registered primary outcome in this study was 7-day PPA at 4 weeks post-QD. This investigation presents findings concerning a range of secondary outcomes, as follows: 7-day PPA at 26- and 52-week follow-up, consecutive 7-day PPA at 26- and 52 weeks follow-up timepoints was also examined, defined as self-reported 7-day PPA (not even a puff of smoke for the past 7 days), and expired-air CO levels less than 5 ppm (among the subset of individuals for whom biochemical verification was conducted), at two or more consecutive timepoints (e.g., weeks 4 and 26, and separately at weeks 4, 26, and 52). Other secondary outcomes included: (1) sustained abstinence, defined as smoking no more than 5 cigarettes from the QD to 26- and 52- week follow-up, respectively; and (2) number of quit attempts at 26- and 52-weeks post-QD.

In addition, we administered a standard demographics questionnaire, Fagerstrom Test for Nicotine Dependence (FTND),^17^ the Smoking Abstinence Self-efficacy Questionnaire,^18^ a 6-item measure used to assess changes in self-efficacy before and after the intervention within different emotional and situational contexts (24-point scale); and the Warwick-Edinburgh mental wellbeing scale (WEMWBS), a 12-item measure used to assess general positive mental health (56-point scale).^19^ Measurements were collected via online questionnaires.^20^

### Statistical Analysis

The sample size was determined based on the requirements linked with the primary pre-registered outcome prior to initiation of the trial: evaluating differences in self-reported 7-day PPA rates at week 4. While the longer term outcomes reported herein are secondary, we made conservative assumptions in our effect size estimation based on prior literature^21^ concerning expected abstinence rates at weeks 26 and 52, and an anticipated 20% attrition rate. According to this set of assumptions, power was set at 80%, with a Type I error rate of 0.05. Statistical analyses were conducted with SAS version 9.4 software (SAS Institute, Cary, NC). To determine smoking abstinence rates, both intention-to-treat (ITT) and per-protocol analyses (PP) were used. Using the ITT approach, all data were analyzed, with unknown smoking status assumed to reflect continued tobacco use.^22^ PP analyses excluded those for whom there were missing or unknown smoking status data and are presented for comparison to ITT results.

Consecutive 7-day point-prevalence abstinence across weeks 4, 26, and 52, based on 7-day PPA at each timepoint, was compared among those who received Quit Genius, relative to the control condition using mixed effects models, controlling for variables known to be associated with smoking cessation treatment outcomes, including gender, race, employment, education, and nicotine dependence severity (indicated by the FTND). Relative Risk ratios (RRs) were used to assess the outcomes for Quit Genius relative to the control group, and chi-square tests were used to test for statistical significance. Comparison of secondary outcome variables between the Quit Genius and control groups was achieved by using two-sample *t* tests for continuous measures and chi-square tests for binary measures.

## Results

### Participants

**Figure 1.**
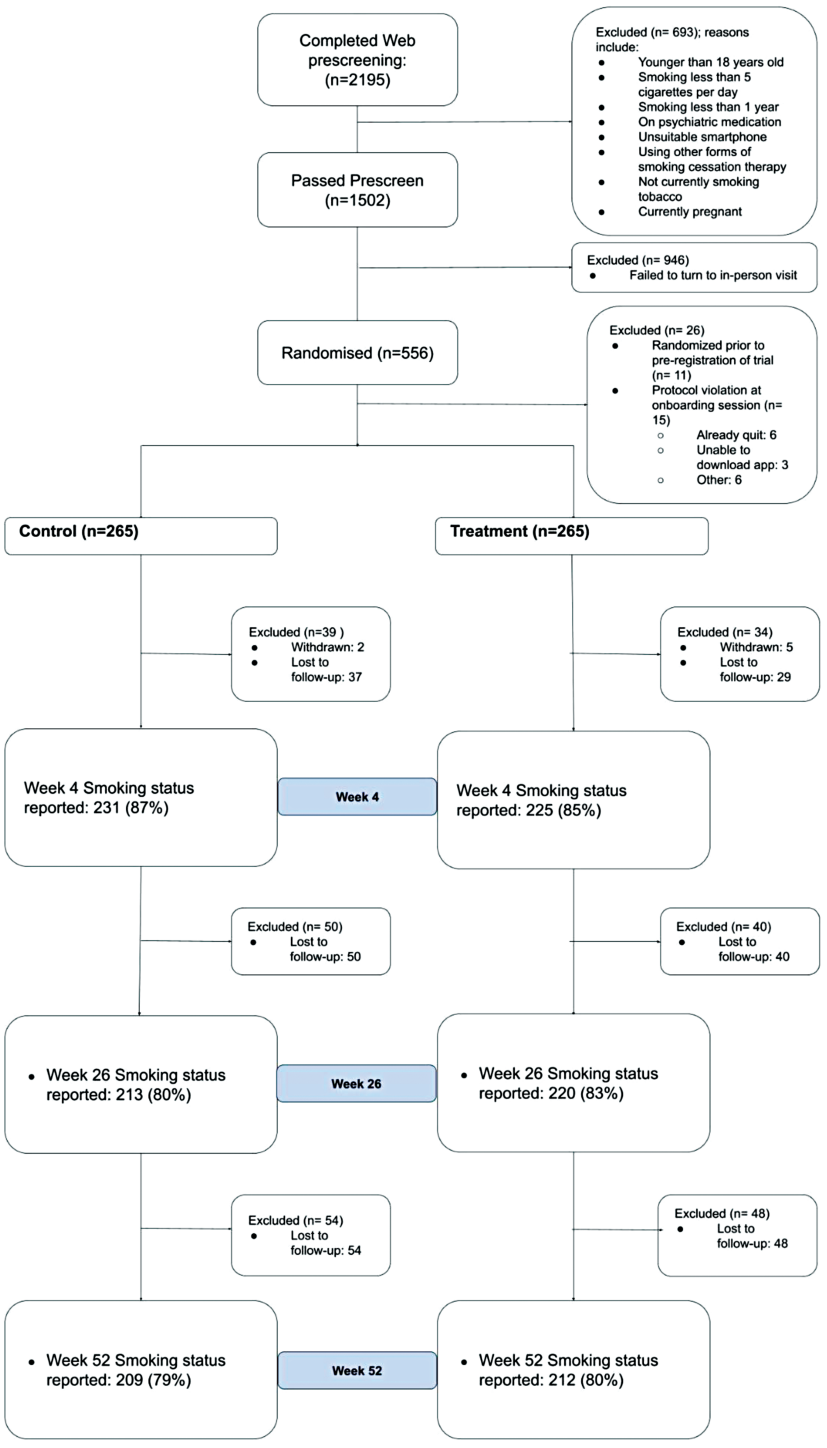
Consort diagram.

The Consort diagram (Figure 1) displays the participant study flow. A total of 2195 individuals were assessed for study eligibility, of which 693 were ineligible. Of the 1502 individuals eligible for inclusion, 946 failed to attend their in-person baseline visit. The remaining 556 participants eligible for inclusion in the study were randomized to study conditions (treatment *n* = 277, control *n* = 279). The ITT analysis included 530 participants (*n* = 265 in each arm; 11 excluded before trial registration, and 15 for baseline protocol violations). Follow-up completion was 81.6.% at 26 weeks, and 79.4% at 52 weeks, with no difference in follow-up rates between study conditions. As shown in Table 1, participants were, on average, 41 (SD = 12) years of age (range: 19–78). The overall sample included 291 (54.9%) men, and was predominantly Caucasian (65.5%), employed (80%), and high school educated. On average, participants reported smoking 14.5 (SD = 7) cigarettes per day, with an average FTND nicotine dependence score of 4 (SD = 2). Most participants (85.0%, *n* = 451) had previously made one or more quit attempts, largely by going cold turkey (48%) or using e-cigarettes (42%) and NRT (30%). There were no significant differences between study conditions in age, ethnicity, educational attainment, gender distribution, or employment status, nor in smoking frequency, nicotine dependence severity, and previous quit attempts.

**Table 1.** Sample characteristics.

	Treatment	Control
Number of participants	265	265
Age (SD)	40 (12)	42 (12)
Female	46.0%	44.0%
Ethnicity		
Caucasian/White	69.0%	62.0%
Black/Caribbean/African/Black	9.0%	11.0%
Asian	7.0%	9.0%
Arab	1.0%	2.0%
Mixed	9.0%	9.0%
Other	3.0%	4.0%
Prefer not to say	2.0%	3.0%
Education		
GCSE or lower	22.0%	23.0%
A-level	25.0%	19.0%
Undergraduate degree	29.0%	29.0%
Postgraduate degree	17.0%	19.0%
PhD	2.0%	1.0%
Prefer not to say	6.0%	9.0%
Employed	79.0%	81.0%
Type of employment (if employed)		
Managerial or professional	60.0%	53.0%
Routine or manual	10.0%	15.0%
Intermediate	10.0%	9.0%
Other	18.0%	19.0%
Prefer not to say	1.0%	3.0%
Cigarettes per day (SD)	14 (6)	15 (7)
Fagerström test for nicotine dependence (range 0–10; SD)	4 (2)	4 (2)
Any past attempt to quit smoking	84.0%	86.0%
Method previously used (if past attempts)		
Cold turkey	47.0%	49.0%
E-cigarettes	42.0%	42.0%
NRT	31.0%	28.0%
Prescription medication	11.0%	15.0%
Smartphone app	9.0%	10.0%
Hypnotherapy	4.0%	7.0%
Psychological therapy	2.0%	2.0%

### Abstinence

The hypothesis that the Quit Genius condition would yield higher rates of self-reported 7-day PPA across the 52-week post-quit period, compared to the control condition was partially supported. Those in the treatment arm were 55% more likely to self-report 7-day abstinence at 4 weeks post-QD compared with those in the control group (risk ratio 1.55, 95% CI 1.23-1.96; *p* < .001; 118/265, 44.5% vs 75/265, 28.3% quit rate). The treatment condition also produced significantly higher self-reported 7-day PPA rates at week 26, relative to the control group (*p* < .01), though this effect did not hold at 52 weeks (*p* > .05). ITT and PP 7-day PPA rates by condition are shown in Table 2.

**Table 2.** Primary and secondary outcomes at 4, 26, and 52 weeks post-quit date

Primary outcome	Treatment	Control	*χ* ^2^		*P*-value	RR	95% CI
7-day point prevalence abstinence 4 weeks (ITT) (*n* = 530) 4 weeks (PP) (*n* = 456)	118 (44.5%) 118 (52.4%)	75 (29.3%) 75 (32.5%)	13.7 17.8	N/A	<.001*** <.001***	1.55 1.62	1.23 to 1.96 1.29 to 2.0
Secondary outcomes	Treatment	Control	*χ* ^2^	*t*-test (*df*)	*p*-value	RR^a^	95% CI
7-day point prevalence abstinence week 26 (ITT) week 26 (PP) week 52 (ITT) week 52 (PP)	95 (35.9%) 95 (44.6%) 92 (34.7%) 92 (44.0%)	73 (27.6%) 73 (33.2%) 78 (29.4%) 78 (36.8%)	4.2 5.9 1.7 2.3		0.03* 0.01* 0.19 0.13		1.32 (1.03 to 1.69) 1.38 (1.08 to 1.75) 1.20 (0.94, 1.54) 1.24 (0.99, 1.57)
Quit attempts beyond initial QD Week 4 Week 26 Week 52	68 (25.6%) 94 (35.5%) 104 (39.2%)	86 (32.6%) 115 (43.4%) 135 (50.9%)	2.6 3.2 6.9	N/A	0.10 0.07 0.009**	0.79 0.82 0.77	0.60 to 1.03 0.66 to 1.01 0.64 to 0.93
Change in self-efficacy Week 4 Week 26 Week 52	4.2 (7.0) 3.8 (8.5) 3.3 (8.6)	3.1 (6.7) 2.0 (6.9) 2.2 (7.8)	N/A	1.7 (527) 2.6 (506) 1.6 (524)	0.09 0.01* 0.12	N/A	1.0 (-0.16 to 2.17) 1.75 (0.42 to 3.07) 1.12 (-0.28 to 2.52)
Change in Mental Well-being Week 4 Week 26 Week 52	0.7 (7.0) 1.4 (8.2) 1.5 (8.4)	0.6 (6.5) 1.0 (7.8) 0.1 (8.3)	N/A	0.2 (525) 0.7 (527) 1.9 (528)	0.83 0.51 0.06	N/A	0.13 (-1.02 to 1.28) 0.45 (-0.90 to 1.81) 1.39 (-0.03 to 2.82)
Consecutive 7-day abstinence Week 26: ITT (*n* = 530) Week 26: PP (*n* = 433) (participant is abstinent at 4 and 26 weeks)	72 (27.2%) 72 (33.8%)	44 (16.6%) 44 (20.0%)	8.7 10.5	N/A	0.003** 0.001**	1.70 1.79	1.22 to 2.37 1.30 to 2.46
Consecutive 7-day abstinence 52 weeks: ITT (*n* = 530) 52 weeks: PP (*n* = 421) (participant is abstinent at 4, 26, and 52 weeks)	60 (22.6%) 60 (28.7%)	35 (13.2%) 35 (16.5%)	8.01 8.9	N/A	0.005** 0.003**	1.71 1.77	1.17 to 2.50 1.22 to 2.54
Sustained abstinence, Week 26, Week 52 (participant ≤ 5 cigs since QD)	73 (27.5%) 58 (21.9%)	40 (15.1%) 30 (11.3%)	11.5 9.9	N/A	<0.001*** 0.002**	1.82 1.93	1.29 to 2.58 1.29 to 2.90

*χ* ^2^ = Chi-squared test; QD, quit date; RR, relative risk ratio; ITT, intention-to-treat analysis; PP, per-protocol analysis.

*p < .05; **p < .01; ***p < .001.

According to ITT analyses, the unadjusted odds of self-reported smoking abstinence over the 52-week course of treatment were significantly higher in the Quit Genius condition, relative to the control group (OR = 4.18, 95% CI = 2.02, 8.64; *p* < .0001). After controlling for gender, demographics, and nicotine dependence severity, the adjusted odds remained significantly higher in the Quit Genius group (OR = 4.16, 95% CI = 2.01, 8.59; *p* < .0001).

**Figure 2.**
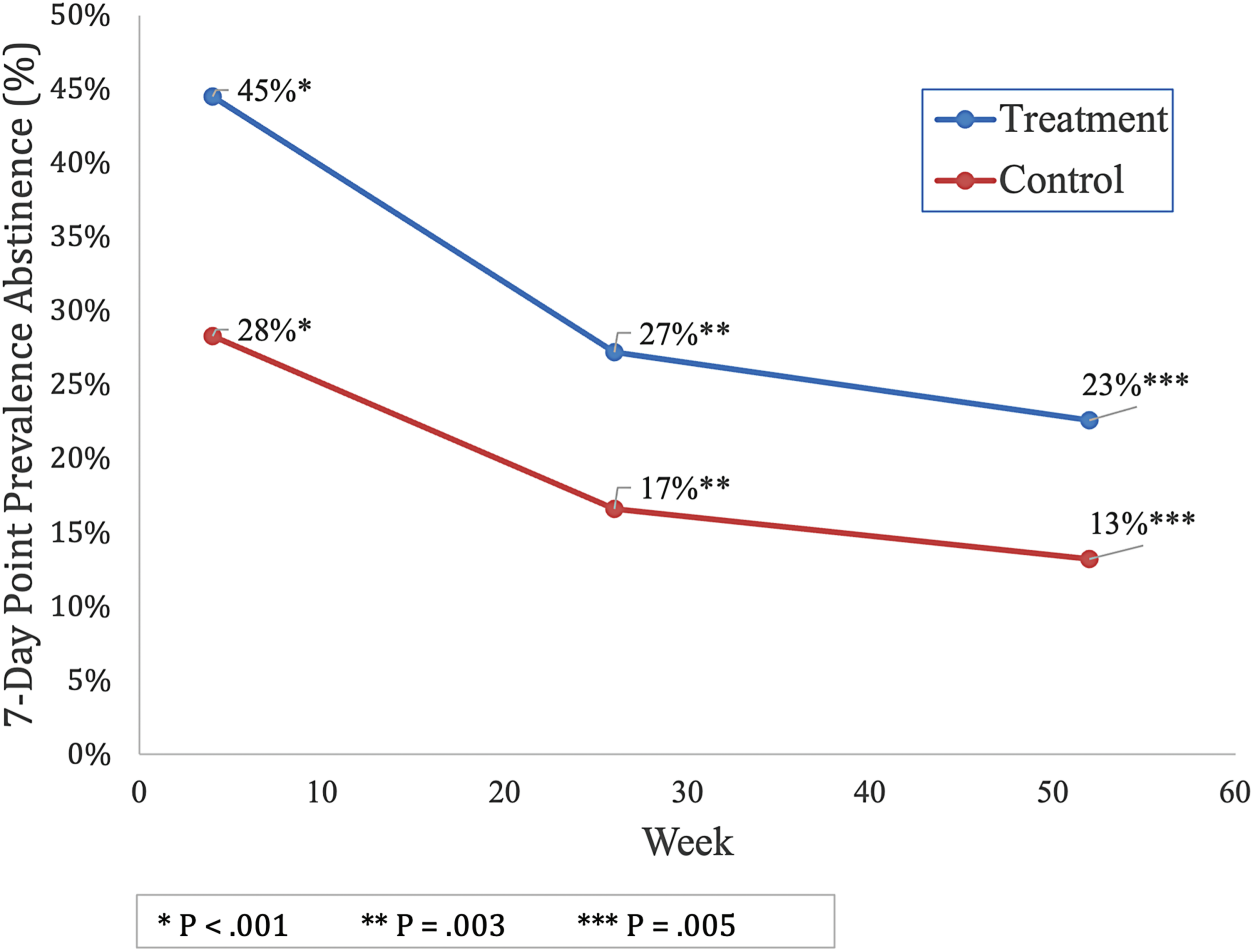
Change in consecutive 7-day point prevalence abstinence.

Likewise, the Quit Genius condition produced higher rates of self-reported consecutive 7-day PPA, relative to controls, across weeks 26 (27.2% vs 16.6%, *p* = .003) and week 52 (22.6% vs 13.2%, *p* = .005) (see Table 2 and Figure 2). Moreover, relative risk ratios indicated that those who received the Quit Genius intervention and had quit successfully at 4 weeks were 70% more likely to self-report 7-day PPA consecutively at week 26 (95% CI = 1.22, 2.37, *p* = .003) and 71% more likely to self-report abstinence through week 52 (95% CI = 1.17, 2.50, *p* = .005).

Quit Genius participants evidenced higher rates of self-reported sustained abstinence compared with those who received the control intervention, both at 26-weeks (RR=1.82, 95% CI, 1.29 -2.58;27.5% vs 15.1% quit rate) and at 52-weeks (RR= 1.93, 95% CI 1.30, 2.91; 21.9% vs 11.3% quit rate).

Among participants who were assigned a CO monitor, 97.1% (*n* = 134) in the Quit Genius condition and 97.9% (*n* = 139) in the control group provided a baseline CO reading. At 4, 26, and 52 weeks post-QD, 88.2%, 81.2%, and 82.5% of self-reported abstainers assigned a CO device provided a CO reading, respectively. Among those reporting abstinence, at 4 weeks post-QD, expired CO <5 ppm corresponded with participant self-report for the majority (93.8%) of participants. At 26 and 52 weeks, self-reported abstinence corresponded with CO <5 ppm in 93.6% and 92.4% of participants, respectively. Moreover, there were no differences observed in quit rates at any of the timepoints among those in the Quit Genius condition who were assigned a CO device, relative to those who were not (*p* = .70 and *p* = .46 at weeks 26 and 52, respectively), nor were any differences in quit rates observed among those in the control group as a function of CO device assignment (*p* = .08 and 0.86 at weeks 26 and 52, respectively).

Though differences in self-efficacy were not observed at week 4 (*p* = .09), a difference emerged at week 26, with larger increases in self-efficacy observed among those who received the Quit Genius intervention, *t*(506) = 2.6, *p* = .01. However, at 52 weeks, that effect had diminished (*p* = .12). No between-group differences were detected in regards to participant mental well-being.

### Engagement

The total number of messages sent to participants by their quit coach averaged 34.7 (SD = 16.7). The average number of app opens was 57.9 (SD = 120.1), and on average participants were actively using the app for 11.8 weeks (SD = 11.5). 42.8% of the Quit Genius participants completed the CBT essentials. The average number of messages sent to a Quit Coach was 15.4 (SD = 22.2) and the number of diary entries made was 13.8 (SD = 29.3). The engagement results indicate that participants who actively engage in the app for 5 to 8 weeks (unadjusted OR = 2.18, *p* = .03; adjusted OR = 2.09, *p* = .05) and those who actively use the app for 9 weeks or more (unadjusted OR = 1.98, *p* = .01, adjusted OR = 2.11, *p* = .07) were more likely to demonstrate consecutive 7-day abstinence at week 52 post-QD compared with those who use the app for less than 5 weeks.

### NRT Use

There were no differences between groups in reported NRT use at each time-point. At 4 weeks, 58.8% (QG; 133) vs 63.2% (Control; 146) *p* = .39; 26 weeks, 34.3% (QG; 73) vs 34.5% (Control; 76) *p* = 1; 52 weeks, 30.6% (QG; 64) vs 29.2% (Control; 62) *p* = .84.

## Discussion

The purpose of this study was to evaluate Quit Genius, a digital clinician-assisted CBT intervention combining pharmacotherapy and behavioral treatment for smoking cessation. Based on the well accepted chronic disease model of addiction,^23^ the 52-week Quit Genius treatment program produced self-reported 7-day PPA rates consecutively at 4, 26, and 52 weeks post-QD that are either comparable to or higher than those associated with more traditional approaches to smoking cessation. Quit Genius participants who effectively quit smoking 4 weeks post-QD were 1.70 times more likely to remain abstinent relative to control group participants at week 26 (27.2% abstinent vs 16.6% abstinent), and 1.71 times more likely to remain abstinent at week 52 (22.6% abstinent vs 13.2% abstinent).

Importantly, these findings extend the preliminary outcomes previously reported,^12^ establishing not only the short-term efficacy of the Quit Genius intervention in producing self-reported smoking abstinence (44.5% at 4 weeks post-QD, according to ITT analyses), but in helping smokers achieve repeated observations of 7-day PPA over the course of 1 year. The intervention approach used in this study differs from others reported in the literature in that it combines psychosocial and pharmacological modalities, and concurrently provides access to the psychosocial component over an extended time period, regardless of participants’ abstinence status over that timeframe. Thus, the current study advances the evidence base for extended, digital health intervention models for smoking cessation. Although PPA rates have been reported across various RCTs of digital health interventions, according to a recent Cochrane review of smartphone and app-based smoking cessation interventions, 6-month abstinence rates ranged from 4% to 18%,^24^ suggesting that the extended, multi-modality approach inherent to the Quit Genius program may contain a combination of elements (i.e., clinician-delivered content, pharmacotherapy, and treatment duration) that optimize the effectiveness of digital, clinician-assisted treatment for individuals with tobacco addiction. Moreover, longer term quit rates reported in the literature typically refer to the proportion of individuals abstinent at a given point in time, rather than capturing consecutive observations of 7-day PPA across multiple follow-ups, a more stringent measurement of favorable treatment outcomes. Thus, while significant variability in intervention components exists between the various digital smoking cessation interventions described in the literature, for which 6-month outcomes range from 6.5% to nearly 30%,^25–28^ the self reported abstinence rates observed in the present study are at the higher end of this range.

Methodological limitations of extant studies, including the use of single-arm designs, and absence of biochemical verification of self-report data, pose challenges to interpretation and generalizability of prior findings. To overcome these limitations, the present study employed a 2-arm parallel-group RCT design with biochemical verification. Despite verifying the CO of a subset of participants, the high correspondence between CO readings in this study and self-reported abstinence is consistent with a review by the Society for Research on Nicotine and Tobacco Subcommittee on Biochemical Verification,^29^ indicating that biochemical validation is not always necessary in smoking cessation studies, because levels of misrepresentation are generally low (0%–8.8%).

There were no significant group differences in quit-attempts at 4 and 26 weeks; however, at 52 weeks, despite having lower self-reported quit rates, control group participants were significantly more likely to have reported additional quit attempts beyond their initial QD than those in the treatment group, suggesting that they continued to attempt, albeit less successfully, to stop smoking. Focusing on re-engaging individuals who initially failed to quit and increasing their motivation to reinitiate quit attempts will likely improve future success rates of Quit Genius.

Self-efficacy is an important process variable underlying successful smoking abstinence outcomes.^30,31^ Improvement in self-efficacy was greater among those who received Quit Genius, relative to controls, an effect that emerged at 26-weeks post-QD. These findings are consistent with prior literature demonstrating extended effects of CBT, which in some cases, yields greater improvement in outcomes after treatment ends, a phenomenon known as a “sleeper effect”,^32^ and extend the increase in reported quit-confidence and self-efficacy observed in Webb et al. (2020) preliminary outcome findings, indicating that digital clinician-assisted CBT interventions can affect similar psychological process variables to face-to-face treatments.^12^ These differences in self-efficacy diminished at 52 weeks post-QD, suggesting that understanding and addressing barriers to maintaining confidence is a potential target for future intervention refinement. Though overall well-being improved generally among participants, there were no group differences in the magnitude of improvement, suggesting that further research is needed to build upon our understanding of how digital interventions can positively target mental wellness among smokers. Nevertheless, given that those receiving psychiatric medication were excluded from participation in this study, the absence of effects on mental well-being may be attributable to a restricted range of mental well-being in the study sample.

Finally, engagement with the Quit Genius program emerged as an important factor in optimizing outcomes, with the extent of engagement, measured according to a combination of factors (i.e., app use, coach interaction, completion of digital CBT). Analyses demonstrated that longer durations of app engagement were predictive of a greater likelihood of long-term cessation extending to 52 weeks post-QD. Though it is unclear which of the indicators of engagement accounted for the greatest variability in outcomes, future research will enable a deeper understanding of the mechanisms of action of digital, clinician-assisted smoking cessation interventions.

### Strengths and Limitations

There are several strengths of this study, including a large sample size, the randomized controlled design, the use of remote biochemical verification and high correspondence between self-reported smoking status and CO measurements, the high participant retention rate and long-term follow-ups.

This study also has several limitations. First, Quit Genius was not compared to another digital intervention. VBA was used as the control intervention due to its frequent use as the first-line intervention for smoking cessation in the UK, and for the participants not allocated a CO device, no mobile-app was provided. Additionally, given the control intervention comprised a single session, in the absence of targeted clinician follow-up, a portion of this group would likely not seek further treatment, possibly reducing its cessation rates. To control fully for time and attention, future studies should consider incorporating a digital intervention without clinician involvement as a comparison group.

Second, this study relied largely on self-reported smoking abstinence status, which may have been exaggerated. To mitigate this, we verified self-reported outcomes using a CO measurement device among 50% of participants in each study condition. Despite the limited biochemical verification data, the consistently high level of agreement between the CO readings and self-reported abstinence across all study timepoints suggests that, in the context of this study, self-report is a reliable indicator for true smoking abstinence. Further, though tests of mediation were not conducted as part of this study, given that a core CBT process change, improvement in self-efficacy, did not have durable effects over the 52 weeks follow-up period, a thorough examination of putative mechanisms of action of the Quit Genius intervention is a logical next step, and efforts to optimize the efficacy of Quit Genius should focus on sustaining changes in self-efficacy over a longer duration. Also, researchers were unblinded to participant group allocation.

Finally, there are some limitations inherent to the study design, as well as the generalizability of the study findings, considering the characteristics of the study sample. In regards to the former, given the components of the Quit Genius intervention that distinguish it from the control condition, coupled with the study design as a 2-arm trial, it was not possible to fully evaluate the relative contribution of these components to key smoking outcomes. Though we found that longer engagement was associated with improved outcomes, it remains unclear whether the primary factor was engagement with the digital CBT, interaction with the quit coach, or the extended treatment model. Future studies disentangling these components are warranted to effectively scale, and understand potential cost-effectiveness of the Quit Genius intervention. Moreover, participants with serious health conditions and/or who were using psychiatric medication were excluded. In light of the high rates of psychiatric comorbidity among smokers, replication and extension of the study to include those with co-occurring mental health conditions will elucidate the utility of Quit Genius across broader, more representative populations of smokers. Additionally, 63% of those initially eligible through online screening did not attend the in-person baseline session. The remaining sample was likely highly motivated to quit smoking, potentially increasing the cessation and retention rates in both arms. Further, given that the study sample comprised a largely urban population, the long term efficacy of a digital health intervention such as Quit Genius among rural groups of smokers, who could benefit tremendously from the accessibility of this approach, remains unknown.

## Conclusion

Quit Genius, a digital clinician-assisted CBT program combining NRT with psychosocial treatment, was effective in achieving smoking cessation at 4, 26, and 52 weeks compared with controls. Still, opportunities exist to improve psychological process outcomes. Studies of the effectiveness of Quit Genius among more diverse populations of smokers are a promising area for future research.

## Supplementary Material

ntac113_suppl_Supplementary_Taxonomy-form

## Data Availability

The data underlying this article will be shared on reasonable request to the corresponding author.
